# Causal systems mapping to promote healthy living for pandemic preparedness: a call to action for global public health

**DOI:** 10.1186/s12966-022-01255-7

**Published:** 2022-02-07

**Authors:** Nicolaas P. Pronk, Mark A. Faghy

**Affiliations:** 1 HealthPartners Institute, 8170 33rd Avenue South, Minneapolis, MN 55425 USA; 2 Department of Health Policy and Management, University of Minnesota, Minneapolis, MN USA; 3 Department of Social and Behavioral Sciences, Harvard T.H. Chan School of Public Health, Boston, MA USA; 4 Human Sciences Research Centre, University of Derby, Derby, UK; 5 Department of Physical Therapy, College of Applied Sciences, University of Illinois at Chicago, Chicago, IL USA

**Keywords:** Physical activity, Nutrition, Obesity, Systems science, Causal mapping, COVID-19, Pandemic, Syndemic

## Abstract

COVID-19 has severely impacted population health and well-being globally. Acknowledging that COVID-19 will not be the world’s last pandemic, improving healthy living factors (i.e., physical activity, healthful nutrition, healthy weight), which are important in mitigating negative outcomes of future infectious disease pandemics, should be prioritized. Although well-documented, promoting healthy living factors remains challenged by a lack of scalability and sustainability due, in part, to a mismatch between intervention focus on individual behavior change as opposed to recognizing complex and multifactorial causes that prevent people from living healthy lifestyles and maintaining them long-term (such as political will, economic benefits, urban planning, etc.). To recognize this complexity in promoting healthy living, we propose the application of systems science methods for the creation of a comprehensive causal systems map of healthy living factors *in the context of* COVID-19 to inform future pandemic preparedness. Generating such a map would benefit researchers, practitioners, and policy makers in multi-sector collaborative efforts to improve public health preparedness in the context of future pandemics in a scalable, sustainable, and equitable manner. This effort should be facilitated by a trusted and widely respected governing body with global reach.

## Introduction

The roles of healthy living behaviors in viral protection have been well-established [1]. For example, habitual physical activity plays a pivotal role in improving immunosurveillance against pathogens and reduces morbidity and mortality from viral infections and respiratory illnesses including influenza, pneumonia, and the common cold [2]. Preliminary data indicate that consistently meeting physical activity guidelines constitutes a significant protective factor against risk for severe coronavirus disease 2019 (COVID-19) related outcomes [3]. Additionally, obesity, a health-related factor directly influenced by physical activity and diet, has shown to be a significant risk factor for adverse COVID-19 related outcomes [4]. Both COVID-19 and obesity have broad global health impacts and are recognized as causal factors in the largest decline in U.S. life expectancy since World War II [5]. Thus, promoting physical activity, healthful nutrition, and healthy living circumstances that mitigate a propensity towards obesity represent necessary actions for endemic and future pandemic preparedness, whilst acknowledging that COVID-19 will not be the world’s last pandemic. Furthermore, the COVID-19 pandemic has heightened public awareness of the need for positive health behaviors, there is an opportunity to leverage the public’s attention and to integrate healthy living behaviors into the contexts of how people live their lives. Ensuring that physical, social, and economic environments support and incentivize healthy living behaviors will make options to engage in such behaviors easier or, perhaps optimistically, the default [6].

Scientific evidence that demonstrates the health benefits of engaging in healthy living behaviors is strong [1–7]. Yet, challenges remain in efforts to translate available evidence of effectiveness into population-level strategies due, in particular, to a lack of intervention scalability and sustainability [7]. Reasons for these limitations relate to a mismatch between an intervention focus on individual behavior change as opposed to recognizing the multifactorial causes of complex system structures and incentives that prevent people from living active lifestyles, such as political will, economic benefits, urban planning, and so forth [7, 8].

The purpose of this paper is to call for a multi-disciplinary and participatory approach to generating a causal systems map of healthy living factors (i.e., physical activity, healthful nutrition, and obesity) *in the context of* COVID-19. The interactions between the pandemics of COVID-19, obesity, physical inactivity, and poor nutrition may be characterized as a syndemic. Successful promotion of and engagement with healthy living factors will significantly impact population preparedness for future pandemics.

## A systems approach

A system is a group of interconnected components and factors with each part influencing the other in various ways that are often not obvious or even anticipated [8]. For example, the human body is a complex system. In turn, it is also a part of larger systems that surround the body and affect it because of influences from many other factors (e.g., physical, social, cultural, economic). Changes in one or more parts of a system affect many other parts—either directly or indirectly. Understanding how systems behave and function allows for identification of the causes of observed phenomena or predict the effects of interventions [8].

Complexity is a key characteristic of a system. Complexity emerges because of the many interactions among the various components of a system [9] and these interactions are not necessarily contained within the separate components. An important characteristic of a complex system is that, once the system is taken apart, its emerging properties are destroyed. For example, one cannot understand cognition (which represents something new and unpredictable that evolves as a result of interactions) by studying a single neuron [8]. The application of systems science to healthy living factors *in the context of* the COVID-19 pandemic has the potential to identify deeper leverage points for more impactful systems change than those operative at the individual behavior change levels.

## Application of systems methods

To help researchers, practitioners, and decision makers (i.e., policy makers) better understand and address complex systems, the application of systems approaches may take different forms. Two major categories include systems mapping (a qualitative method) and systems modeling (a quantitative method) [8, 9]. Mapping helps researchers and decision-makers to better “see” a system by developing a diagram or other type of visualization of relevant system components and connections among them. Systems maps can elucidate what is currently known and not known about a system, what mechanisms of action are involved, what additional data and studies may be needed, and how best to prioritize among many factors to stimulate change. A systems map can also be used to identify and make visible the context of important components, interactions, and leverage points of a complex system [8].

Whereas systems mapping can show the “framework” of a system, systems modeling goes several steps further by representing how the system operates quantitatively across time and space and can test and evaluate different policies and interventions. Systems mapping often precedes systems modeling, setting up a rough blueprint or outline that can be used in systems modeling that will apply mathematical equations or computational algorithms to represent the components, relationships, processes, and interactions among the various components of a system [8].

The process of building systems maps is just as important as the outcome of a map itself [8]. Representation and inclusion of key stakeholders ensures their voices and perspectives are integrated and reflected in the mapping exercise and can bring various parties closer together. Engagement in the process of building a map can bring consensus for prioritization. Benefits of a transparent multi-stakeholder process of systems mapping include explaining natural phenomena from multiple perspectives, challenging prevailing wisdom, demonstrating trade-offs, and provoking new research or policy questions—when considering the weighty matters of preserving and safeguarding health and function from pandemic threats, these are worthy objectives to pursue.

## Context matters

Previous work has recognized the potential that systems approaches to healthy living behavior promotion may bring. For example, specific to physical activity, a systems framework was introduced by the World Health Organization Global Action Plan on Physical Activity [10]. Examples of system maps for physical activity, nutrition, or obesity may be identified across several literatures. These frameworks visually represent broad but interconnected considerations such as transport, societal, sociopolitical, sector-specific, individual, and biological factors. The maps are also useful to identify and make explicit the potential mechanisms that may drive improvement in each of these factors separately. However, existing maps lack the context of multiple healthy living factors interacting with the severe shocks of the COVID-19 pandemic to systems that affect people’s lives in overarching and myriad ways. Habitual physical activity, healthful nutrition, and healthy weight status are recognized as strong preventive factors with physical, mental, emotional, social, and economic benefits—all systems impacted by COVID-19 at both individual and population levels. To promote healthy living behaviors and factors for pandemic preparedness in a scalable and sustainable way, there is a need to map healthy living factors in the context of the COVID-19 pandemic and consider their interactivity as an emerging syndemic.

Questions such as what are the dynamic consequences of the COVID-19 pandemic for people with low levels of habitual physical activity, poor nutrition, or obesity and how do these consequences intersect? How are various factors affecting the accumulation (or stock) of healthy living factors as disaggregated by population demographics (e.g., racial/ethnic social groups, children, and adults)? What is the role of healthy living in mitigating the effects of long-term impacts of COVID-19 on people’s health and well-being? Where in this system are the most important places to intervene in a manner that is at once scalable, sustainable, and equitable? Benefits of this approach would relate to post-pandemic actions generating higher levels of population health and well-being.

## Developing a healthy living causal and solutions system map for pandemic preparedness

We propose a call-to-action for the promotion of healthy living factors for post-pandemic preparedness by advocating for a multi-stakeholder and global forum to develop a physical activity-nutrition-obesity causal systems map using COVID-19 as an example of a severe shock to social systems. Well-respected, trusted institutions with global reach should facilitate a multi-stakeholder process to produce a systems map reflecting causality and upon which currently available evidence-based solutions for healthy living promotion may be superimposed and gaps may be identified. This effort would require thorough reviews of current literatures and follow formal systems methods. The process should be transparent, inclusive, comprehensive, and open to validation. Following completion of such a systems map, we would advocate for modeling efforts to iterate and improve upon this initial version and generate opportunities for research and innovations into interventions designed to achieve population reach and sustain improvements over time.

## Conclusion

The broad and severe health and wellbeing impacts and outcomes of the COVID-19 pandemic can be lessened and to some extent prevented with higher levels of habitual healthy living practices. Intervention approaches must consider the role large systems play in creating environments that influence behavior. Systems mapping may help to identify deep leverage points that are critical to effective systems change. We propose a call-to-action for a global public health to establish a system map for healthy living promotion for pandemic preparedness.

## Data Availability

Not applicable.
